# Focal adhesion kinase inhibition synergizes with nab-paclitaxel to target pancreatic ductal adenocarcinoma

**DOI:** 10.1186/s13046-021-01892-z

**Published:** 2021-03-09

**Authors:** T. Y. S. Le Large, M. F. Bijlsma, B. El Hassouni, G. Mantini, T. Lagerweij, A. A. Henneman, N. Funel, B. Kok, T. V. Pham, R. de Haas, L. Morelli, J. C. Knol, S. R. Piersma, G. Kazemier, H. W. M. van Laarhoven, E. Giovannetti, C. R. Jimenez

**Affiliations:** 1 Department of Surgery, Cancer Center Amsterdam, Amsterdam University Medical Centers, VU University Amsterdam, Amsterdam, The Netherlands; 2 Department of Medical Oncology, Cancer Center Amsterdam, Amsterdam University Medical Centers, VU University, De Boelelaan 1117, 1081 HV Amsterdam, The Netherlands; 3 Laboratory for Experimental Oncology and Radiobiology, Cancer Center Amsterdam, Amsterdam University Medical Center, University of Amsterdam, Amsterdam, the Netherlands; 4 OncoProteomics Laboratory, Department of Medical Oncology, Cancer, Cancer Center Amsterdam, Amsterdam University Medical Centers, VU University, De Boelelaan 1117, 1081 HV Amsterdam, The Netherlands; 5 Oncode Institute, Amsterdam, The Netherlands; 6 Cancer Pharmacology Lab, AIRC-Start-Up, Fondazione Pisana per la Scienza, Pisa, Italy; 7 Department of Neurosurgery, Cancer Center Amsterdam, Amsterdam University Medical Centers, VU University Amsterdam, Amsterdam, The Netherlands; 8 Azienda Ospedaliero-Universitaria Pisana, Pisa, Italy; 9 Department of Medical Oncology, Cancer Center Amsterdam, Amsterdam University Medical Center, University of Amsterdam, Amsterdam, the Netherlands

**Keywords:** Pancreatic cancer, Therapy, FAK, EPHA2, MET, Phosphoproteomics

## Abstract

**Background:**

Pancreatic ductal adenocarcinoma (PDAC) is a very lethal disease, with minimal therapeutic options. Aberrant tyrosine kinase activity influences tumor growth and is regulated by phosphorylation. We investigated phosphorylated kinases as target in PDAC.

**Methods:**

Mass spectrometry-based phosphotyrosine proteomic analysis on PDAC cell lines was used to evaluate active kinases. Pathway analysis and inferred kinase activity analysis was performed to identify novel targets. Subsequently, we investigated targeting of focal adhesion kinase (FAK) in vitro with drug perturbations in combination with chemotherapeutics used against PDAC. Tyrosine phosphoproteomics upon treatment was performed to evaluate signaling. An orthotopic model of PDAC was used to evaluate the combination of defactinib with nab-paclitaxel.

**Results:**

PDAC cell lines portrayed high activity of multiple receptor tyrosine kinases to various degree. The non-receptor kinase, FAK, was identified in all cell lines by our phosphotyrosine proteomic screen and pathway analysis. Targeting of this kinase with defactinib validated reduced phosphorylation profiles. Additionally, FAK inhibition had anti-proliferative and anti-migratory effects. Combination with (nab-)paclitaxel had a synergistic effect on cell proliferation in vitro and reduced tumor growth in vivo.

**Conclusions:**

Our study shows high phosphorylation of several oncogenic receptor tyrosine kinases in PDAC cells and validated FAK inhibition as potential synergistic target with Nab-paclitaxel against this devastating disease.

**Supplementary Information:**

The online version contains supplementary material available at 10.1186/s13046-021-01892-z.

## Background

Pancreatic ductal adenocarcinoma (PDAC) is an unsolved major health problem, with only 9% of patients alive 5 years after diagnosis [1]. Surgery offers the only curative treatment, but most patients present with advanced disease, at which point palliative chemotherapy is the only option to slow disease progression. First-line treatment with combinations of chemotherapeutics, such as FOLFIRINOX and nab-paclitaxel with gemcitabine, has improved survival in the last few years [2, 3]. However, second- and third-line treatment options are not standardized and new therapies are warranted. Targeting aberrantly activated tyrosine kinases by tyrosine kinase inhibitors (TKIs) has proven successful in several other solid tumors, for example targeting the mutated epithelial growth factor receptor (EGFR) in non-small cell lung carcinoma [4], or mutated Serine/threonine-protein kinase B-raf in melanoma [5]. However, similar successes have not been achieved to date in PDAC.

The most prominent driver mutation in PDAC is *KRAS*, which is present in up to 90% of tumors [6]. Despite efforts to develop a molecule or therapeutic strategy that can effectively inhibit oncogenic RAS signaling, it is unlikely that there will be a “one-size-fits-all” drug for RAS-driven PDAC soon [7, 8]. The typical panel of mutated genes present in PDAC is further completed by inactivating mutations in *TP53*, *SMAD4* and *CDKN2A* [9, 10].

Lack of targetable mutations warrants new strategies unravelling functional proteins and post-translational modifications in order to identify new key pathways and thus potential drug targets. One of such promising strategies is to investigate the activity of signaling pathways that drive PDAC tumor biology by evaluating cell signaling driven by protein phosphorylation. This process is a tightly regulated complex series of processes in normal cells, but is often deregulated in cancer cells [11]. Remarkably, elucidation of tumor biology and aberrant cellular signaling networks requires an approach that captures protein activation on a global scale. Mass spectrometry-based phosphoproteomics can provide this information on the activation of individual proteins and their associated pathways, thus expanding the reach of genomics and transcriptomics analyses [12–14]. Therefore, this metric has received increasing attention as a novel approach to reveal the signaling that drive cancer growth, and to predict tumor prognosis and/or drug resistance [13, 14].

In the present study, we aimed to utilize phosphotyrosine phosphoproteomic analyses to uncover aberrant pathways in PDAC. Interestingly, despite a high degree of heterogeneity at the genetic and transcriptomic level, many commonly activated pathways driving our PDAC models were observed. Among these kinases the focal adhesion kinase (FAK) emerged as the most interesting target, suggesting a novel therapeutic option in combination with known chemotherapeutics for the treatment of PDAC patients.

## Material and methods

### Cell culture

For this study, eleven pancreatic cancer ATCC cell lines were evaluated for their phosphoproteome. Cell lines were authenticated via STR analysis, and tested negatively for mycoplasma monthly. AsPC1, BxPC3, CFPAC1, HPAC, HPAF-II, PANC-1, PL45, MIA PaCa-2 and Suit-2 were cultured in RPMI medium (Lonza, Switzerland) supplemented with 8% FBS (Biowest, France) and 1% penicillin and streptomycin (Lonza). Capan-2 and Hs766t were cultured in supplemented DMEM medium (Lonza). An immortalized pancreatic ductal cell line HPDE was kindly supplied by dr. Tsao [15] and cultured in supplemented KGM medium (Lonza). Five primary cell lines (PDAC 1–5), previously established [16], were cultured in supplemented RPMI medium. Cells were maintained at 37 °C and 5% CO_2._

### Cell lysates and phosphopeptide enrichment

To evaluate phosphorylation status, cell lysates were prepared in 9 M Urea buffer supplemented with phosphatase inhibitors, phosphopeptides (PP) were enriched according to protocols established previously [17, 18]. Briefly, lysates were created from cells cultured at 70% confluency and subsequently sonicated. The BCA method (ThermoPierce, USA) was performed to determine protein concentration. A total of 10 mg of protein was used for digestion with phosphotyrosine enrichment. A control lysate of HCT116 (colon carcinoma cell line) was used as benchmark sample.

In-solution digestion was performed with Sequencing Grade Modified Trypsin (Promega, USA). Next, peptides with a phosphorylated tyrosine residue were enriched with immunoaffinity beads against phosphotyrosine peptides (PTMScan Phospho-Tyrosine Rabbit mAb (P-Tyr-1000) kit #8803, Cell Signaling, USA). Phosphopeptides were desalted with 20 μl StageTips and eluted into glass-lined autosampler vials.

### Whole-in-gel digestion

For in-depth identification of protein expression, lysates from our five primary cell lines were evaluated using our previously validated protocol [19]. Briefly, 50 μg protein was loaded on 4–12% gradient NuPage gels (Thermofisher, USA). Proteins were stained with Coomassie Brilliant Blue, subsequently reduced and alkylated. Gel bands of each sample were divided into 5 fractions and digested with trypsin overnight. Peptides were extracted from the gels with formic acid / acetonitrile solutions and were stored until measurement.

### Nano-LC-MS/MS and protein identification

Peptides were separated by an Ultimate 3000 nanoLC-MS/MS system (Dionex LC-Packings, the Netherlands) equipped with a 40 cm × 75 μm ID fused silica column custom packed with 1.9 μm 120 Å ReproSil Pur C18 aqua (Dr Maisch GMBH, Germany). After injection, peptides were trapped at 6 μl/min on a 10 mm × 100 μm ID trap column packed with 5 μm 120 Å ReproSil Pur C18 aqua at 2% buffer B (buffer A: 0.5% acetic acid (Fischer Scientific), buffer B: 80% ACN, 0.5% acetic acid) and separated at 300 nl/min in a 10–40% buffer B gradient in 90 min (120 min inject-to-inject) at 35 °C. Eluting peptides were ionized at a potential of + 2 kVa into a Q Exactive mass spectrometer (Thermo Fisher, Germany). Intact masses were measured at resolution 70.000 (at m/z 200) in the orbitrap using an AGC target value of 3 × 10^6^ charges. The top 10 peptide signals (charge-states 2+ and higher) were submitted to MS/MS in the HCD (higher-energy collision) cell (1.6 amu isolation width, 25% normalized collision energy). MS/MS spectra were acquired at resolution 17.500 (at m/z 200) in the orbitrap using an AGC target value of 2 × 10^5^ charges and an underfill ratio of 0.1%. Dynamic exclusion was applied with a repeat count of 1 and an exclusion time of 30 s.

### Protein identification

MS/MS spectra were searched against the uniprot human reference proteome 2014_01_NO_fragments FASTA file (61,552 entries) using MaxQuant [20] 1.5.2.8 (protein expression) and the 2015_08_NO_fragments FASTA file (62,447 entries) (phosphoproteomics). Enzyme specificity was set to trypsin and up to two missed cleavages were allowed. Cysteine carboxamidomethylation (Cys, + 57.021464 Da) was treated as fixed modification and serine, threonine and tyrosine phosphorylation (+ 79.966330 Da), methionine oxidation (Met, + 15.994915 Da) and N-terminal acetylation (N-terminal, + 42.010565 Da) as variable modifications. Peptide precursor ions were searched with a maximum mass deviation of 4.5 ppm and fragment ions with a maximum mass deviation of 20 ppm. Peptide, protein and site identifications were filtered at an FDR of 1% using the decoy database strategy. The minimal peptide length was 7 amino-acids and the minimum Andromeda score for modified peptides was 40 and the corresponding minimum delta score was 6. Proteins that could not be differentiated based on MS/MS spectra alone were grouped to protein groups (default MaxQuant settings). Phosphopeptide identifications were propagated across samples using the match between runs option checked. Protein expression searches were performed with the label-free quantification option selected.

### Label-free phosphopeptide quantification

Phosphopeptides were quantified by their extracted ion intensities (‘Intensity’ in MaxQuant). For each sample the phosphopeptide intensities were normalized on the median intensity of all identified phosphopeptides of the dataset (‘normalized intensity). Data was measured in two datasets and were normalized between datasets with quantile normalization after removal of failed samples.

### Pathway analysis

Data was analyzed for biology with single-sample gene set analysis (ssGSEA) [21] of KEGG pathways with R(Version 3.5.2). Phosphorylation networks were further evaluated with the phosphopath plugin of Cytoscape [22]. INKA analysis [23] was performed to identify downregulation of inferred kinase activity upon drug perturbations.

### Western blot analysis

Validation of proteins and phosphorylation sites were performed by SDS-PAGE Western blot analysis (WB). Following electrophoresis on 10% acrylamide gels, 20 μg of protein was transferred to nitrocellulose membranes. These were blocked with 3% milk in PBS/Tween-20 and incubated with 0.1% diluted primary antibody (FAK Antibody Sampler Kit #9330, ACTB # 4970S, Cell Signaling) and 0.05% secondary antibody (anti-rabbit HRP # 7074S, Cell signaling) in blocking buffer. Protein was visualized with SuperSignal West Pico ECL substrate (Thermofisher) on a UVITEC Imaging System (Uvitec Ltd., UK).

### Drugs and drug perturbation experiments

Drugs against identified possible targets against PDAC were evaluated. Defactinib (Axon Medchem BV, Groningen), TAE226 (Selleckchem), VS-4718 (Selleckchem) and paclitaxel (Sigma) were dissolved in DMSO. Gemcitabine (kindly provided by Eli Lilly Corporation, USA) was dissolved in water. Nab-paclitaxel (Celgene, USA) was dissolved in 0.9% NaCl. 72-h drug perturbation assays were performed with a previously established protocol [16]. In short, 3000–7000 cells were plated per well in a 96-well plate (Greiner, Austria). After attachment overnight, cells were treated with a concentration-range (0–10 μM) for 72-h. After treatment, proteins of viable cells were precipitated with TCA and stained with Sulforhodamine B (SRB). Cell growth was evaluated compared to control wells treated with vehicle (DMSO). For the combination treatment, dissolved defactinib was added either at the inhibitory concentration (IC) of 25 or 50%. The second drug was added in the original drug range (paclitaxel 0–100 nM, gemcitabine 0–1250 nM). The effectiveness of the combination treatment was compared to monotreatment by the median drug effect analysis method. The combination index (CI) was calculated with CalcuSyn software (Biosoft, UK). A CI of below 0.8 indicates a synergetic cytotoxic effect. A CI between 0.8 and 1.2 indicates additive effect and above 1.2 indicates antagonistic effect of the combination therapy.

### Drug accumulation

PDAC-1 cells treated in vitro with paclitaxel, defactinib, vehicle or the combination were used for evaluation of intracellular drug concentrations after 2 h and 24 h exposures. Cells were homogenized and used for concentration measurement as described previously [24]. Briefly, cell pellets were suspended in 100 μl of water and measured for protein content using the Pierce BCA protein kit (Thermo Fischer Scientific BV, the Netherlands). An aliquot of 20 μl was then extracted with 80 μl of acetonitrile, centrifuged at 2575 g for 10 min at 4 °C, before 1 μl of the supernatant was injected onto a validated LC-MS/MS system to determine defactinib and paclitaxel concentrations.

### In vitro migration assay

The effect of treatment on the capability of PDAC cells to migrate was evaluated in vitro as described before [25]. Cells were plated in a high density in 96-well plates and were allowed to attach overnight. Subsequently, a stable scratch was created in the cell layer in each well. Next, cells were exposed to drugs for 24 h (defactinib 1 μM or DMSO). Images were taken at exposure (time 0), and subsequently at 6 h, 20 h, and 24 h. Average wound closure percentage was calculated per well and treatment group.

### Microtubule polymerization analysis

PDAC cells were treated with paclitaxel (200 ng/ml), defactinib (1 μM) or the combination for 2 h. Cells were prepared for FACS analysis as described previously and stained with anti-tubulin-FITC conjugated antibody (1:50, Cell Signaling) [26]. Cells were analyzed by flow cytometry on a FACSCalibur (Becton Dickinson, Franklin Lakes, USA).

### Mice experiments

Animal experiments were approved by the Committee on Animal Experiments of the VU University Amsterdam, the Netherlands. For the orthotopic pancreatic cancer model, primary cells (PDAC-1) were transduced with a lentiviral vector encoding firefly-luciferase as described previously [16]. The pancreas of 6–8-weeks-old living female athymic mice (Envigo) were injected with 1 × 10^6^ cells in a volume of 10 μl PBS. To measure tumor growth, bioluminescence imaging (BLI) was used. For this, 150 μl dissolved D-Luciferin (Sigma) was injected intraperitoneally (ip) and mice were anesthetized with isoflurane (2.5%, Sigma) and imaged with a Xenogen-IVIS Lumina System (Xenogen Corp., USA). Tumor corrected for background BLI. Four days after tumor inoculation, BLI was measured in the cohort and the mice were stratified into 4 groups with equal BLI averages. One week after inoculation, treatment was started twice a day with 25 mg/kg defactinib diluted in 0.5% methylcellulose 0.1% Tween-80 per os (po), 1-10 mg/kg nab-paclitaxel intravenously (iv), or vehicles (0.9% NaCl intravenously (iv) and/or 0.5% methylcellulose 0.1% Tween 80 po). Mice were treated for 2 cycles of 5 days treatment of defactinib, with addition of a total of 31 mg/kg nab-paclitaxel in 4 doses during 2 weeks. Tumor growth was evaluated bi-weekly. Mice were sacrificed upon reaching of humane end point.

### Statistical analysis

In vitro validation studies were replicated at least three times. Data are expressed as means ± standard-error of the mean (SEM). Data were analyzed by (un)paired Student’s t-test or the non-parametric Mann-Whitney test. Outlier analysis was performed with Grubbs test. Tumor growth rate during treatment was calculated as the slope of the growth and tested by the F-test. Tumor size was evaluated per measurement and tested by two-way ANOVA. Overall survival was visualized with Kaplan-Meier curves and evaluated by log-rank test. Pharmacokinetic data are expressed as median ± standard deviation (SD).

## Results

### Phosphoproteomic analysis reveal abundant phosphorylated kinases in PDAC

**Fig. 1:**
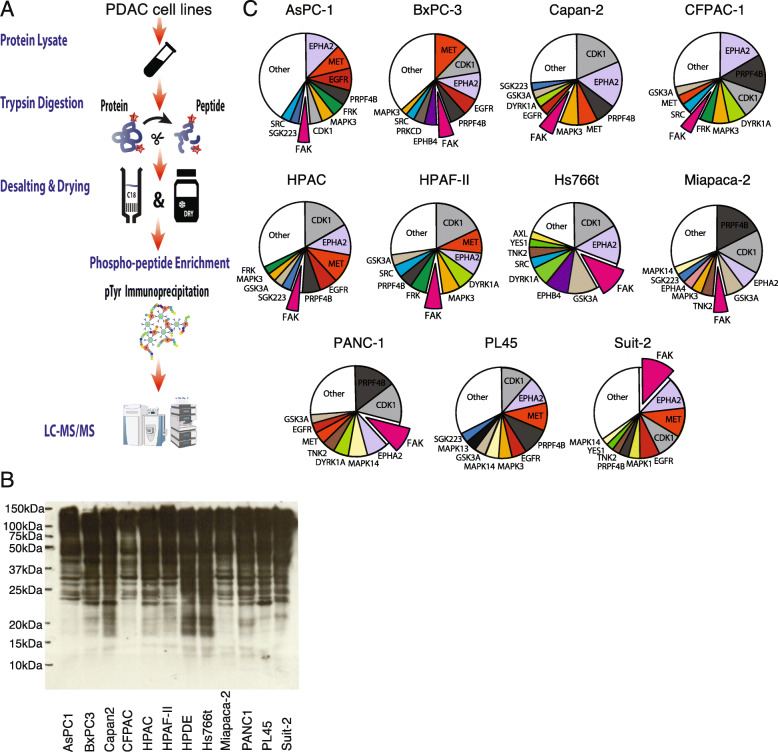
Global analysis of the phosphoproteome of PDAC cell lines shows abundant kinase phosphorylation. **a.** Workflow of analysis. **b.** Western blot analysis of cell line panel proteins containing phosphorylated tyrosine aminoacids. **c.** Pie charts portrait the ranking of identified phosphorylated kinases in each PDAC cell line

As the cellular signaling that drives cancer depends largely on the aberrant phosphorylation of kinases, the phosphoproteome of a panel of cell lines of PDAC incorporating the full heterogeneity of the disease was analyzed after enrichment for phosphotyrosine peptides (pY). The workflow of this analysis is presented in Fig. 1a. The cell lines were evaluated for general tyrosine phosphorylation and showed evident abundance of phosphotyrosine-containing proteins on multiple molecular weights (Fig. 1b). Biological and technical replicates showed high correlation (Supplemental Fig. 1A). A total of 2685 phosphopeptides, derived of 1237 unique phosphoproteins were identified. On average 406 (range: 247–602) phosphoproteins per sample were identified. The data include a wide range of the human kinome [27], including 60% of the known human receptor tyrosine kinases (RTKs) and 68% of the known non-receptor tyrosine kinases (non-RTKs, Supplemental Fig. 1B). Unsupervised clustering of phosphoproteins showed a variance in the level of phosphorylation between cell lines (Supplemental Fig. 1C). Interestingly, several kinases were highly expressed in all samples (Fig. 1c). For RTKs, EGFR, MET and EPHA2 were phosphorylated in almost all cell lines, highlighting a pattern of multiple cancer pathway activation. Of the non-RTKs, FAK was phosphorylated in all models. Some variance of contribution of each of the top phosphorylated kinase was seen between cell lines, but not many outliers were identified when evaluating the contribution of a phosphorylated kinase to the phosphorylated kinase palette of each cell line. In Suit-2, FAK was identified as an outlier (Supplemental Fig. 2A).

### FAK is activated in PDAC cells and its inhibition has strong antitumoral efficacy

**Fig. 2:**
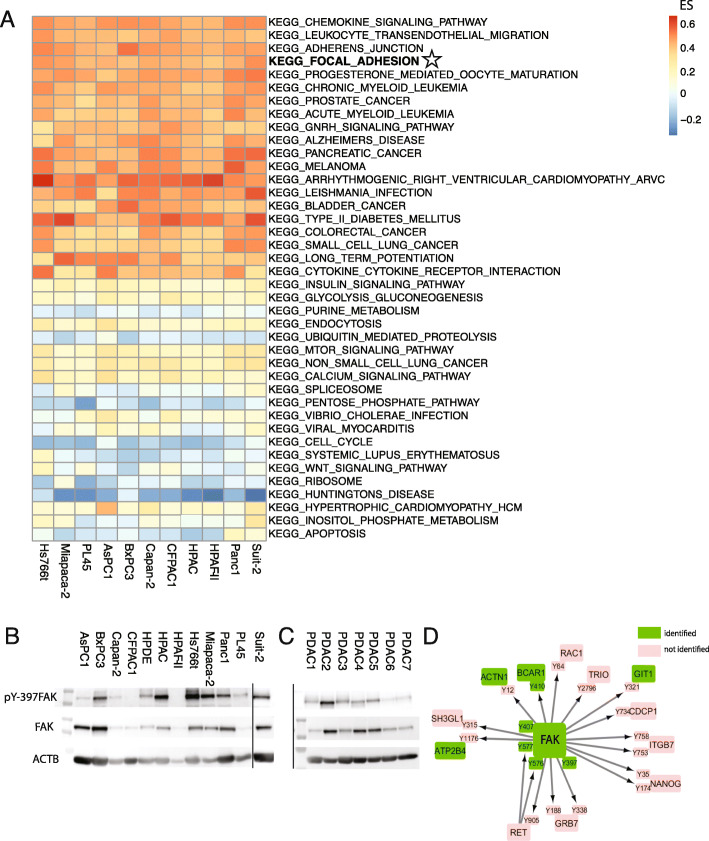
Biological evaluation of activated pathways in PDAC cell lines identifies FAK. **a.** ssGSEA of phosphorylated proteins highlight focal adhesion pathway in all cell lines. Heatmap shows enrichment score (ES) of top 20 up and downregulated KEGG pathways (coloring based on ES, red = upregulated, blue = downregulated). **b.** Western blot of FAK expression and phosphorylation of tyrosine-sites validates phosphoproteome analysis (loading control ACTB). **c.** Western blot of FAK in primary cell line panels confirms abundant expression in other PDAC models (loading control ACTB). **d.** Kinase-substrate relations of FAK are visualized in an interactive network of cell line Suit-2, which represents the highest relative FAK phosphorylation (visualization by Phosphopath [22])

To delineate the activated pathways that drive PDAC, single-sample GSEA was performed on the phosphoproteome data using KEGG Pathways as gene sets (Fig. 2a). Several cancer-related pathways were found to be enriched. Interestingly, “Focal adhesion” and “Adherence junction” was enriched in all cell lines. We evaluated the quantitative phosphoproteome data for this kinase. Indeed, phosphorylated FAK was present in all PDAC cell lines (Supplemental Fig. 2B), including phosphorylation of multiple of its activation sites. Network analysis of downstream substrates showed functional phosphorylation of multiple substrates in agreement with FAK activity (Fig. 2d). To validate these results, WB analysis was performed. Phosphorylation of FAK on its regulatory site pY-397 could be validated in most cell lines (Fig. 2b) and showed levels corresponding with the quantitative mass spectrometry phosphoproteome analyses except for HPAF-II. To further evaluate our findings, primary cell cultures from PDAC tumors were evaluated. Indeed, these cells also showed FAK phosphorylation underscoring the ubiquity of this kinase activation in PDAC (Fig. 2c).

**Fig. 3:**
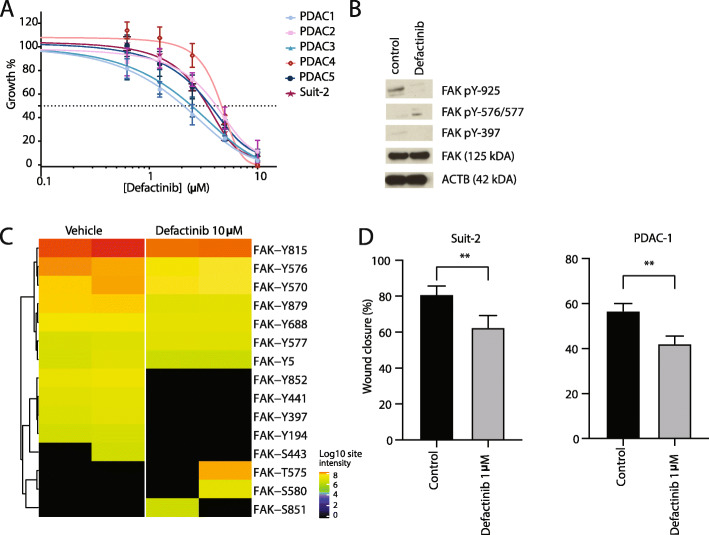
FAK inhibition by TKI defactinib hampers tumor properties of PDAC cells. **a.** Treatment with defactinib reduced PDAC cell proliferation (averages with corresponding SEM are visualized). **b.** Western blot analysis after 2 h of exposure to 10 μM defactinib showed reduced phosphorylation of activation site pY-397 and pY-925, but not pY-576/577. **c.** Heatmap of FAK p-site log intensities after defactinib drug perturbation showed downregulation of multiple p-sites of FAK including regulatory site pY-397 (red = upregulated, green = downregulated, black = not detected). **d.** Migration at 24 h assessed by scratch assays was significantly hampered by treatment with 1 μM defactinib in Suit-2 and primary cell line PDAC1 (Error bars depict SEM of at least 3 biological replicates)

FAK is a protein with a variety of functions in cell biology, and is a central node connecting multiple pathways including integrin- and RTK-signaling [28]. Since high FAK activity was observed in the phosphoproteome, pathway and substrate analyses, we explored the efficacy of multiple (pre)clinical FAK inhibitors against the cell line Suit-2, in addition to the primary cell line panel. Three FAK inhibitors (defactinib, VS-4718 and TAE-226) were evaluated in vitro. IC50s ranged within 2.0–5.0 μM for defactinib (Fig. 3a), 1.8–5.2 μM for VS-4718 and 1.0–1.6 μM for TAE226 (Supplemental Fig. 2C, Supplemental Fig. 2D), demonstrating the efficacy of FAK inhibition against PDAC proliferation. Defactinib is the leading anti-FAK drug currently under investigation in multiple clinical trials against solid tumors [28–31]. Therefore, further functional experiments were conducted with this compound to provide translational relevance and apt translation to clinical use.

The target selectivity of FAK by defactinib was shown in a database of chemical proteomics [32]. It functions via competition of the ATP-binding site of FAK, reducing the enzymatic effect of FAK by inhibition of phosphorylation of pY-397 and downstream activation [33]. Upon exposure of PDAC cells to this drug, after 2 h of exposure, dephosphorylation of the regulatory site pY-397 and functional site pY-925 was seen (Fig. 3b). Phosphorylation of pY-576/577 was not inhibited by two hours of exposure to defactinib as detected by western blot. To confirm FAK enzymatic inhibitory effect, we performed phoshoproteomic analysis of cells after exposure to the drug defactinib. Importantly, multiple phosphosites of FAK showed significant down regulation (Fig. 3c), including pY-397, the activation loop and other functional phosphosites of FAK. Additionally, a broad downregulation of phosphorylation of multiple phosphoproteins and kinases were seen (Supplemental Fig. 3A, Supplemental Fig. 3B). This is consistent with the broad role of FAK in intracellular signaling. Evaluation of kinase activity with INKA showed pronounced inhibition of FAK activity (Supplemental Fig. 3C). Additionally, phosphorylation of several interactors of FAK identified in Fig. 2d were significantly reduced by defactinib treatment (Supplemental Fig. 3D), highlighting a reduced signaling cascade upon treatment. Specifically, protein BCAR1 had multiple reduced phosphopeptides upon defactinib treatment. This protein is associated with cell adhesion and migration [34]. Moreover, FAK itself contributes to cell motility and invasion [35]. Indeed, we observed that the migration of cells was hampered in vitro by exposure with defactinib (Fig. 3d), underscoring the multimodal therapeutic effect of this treatment.

### Combination of FAK-inhibitor with paclitaxel shows synergy against PDAC cells in vitro

**Fig. 4:**
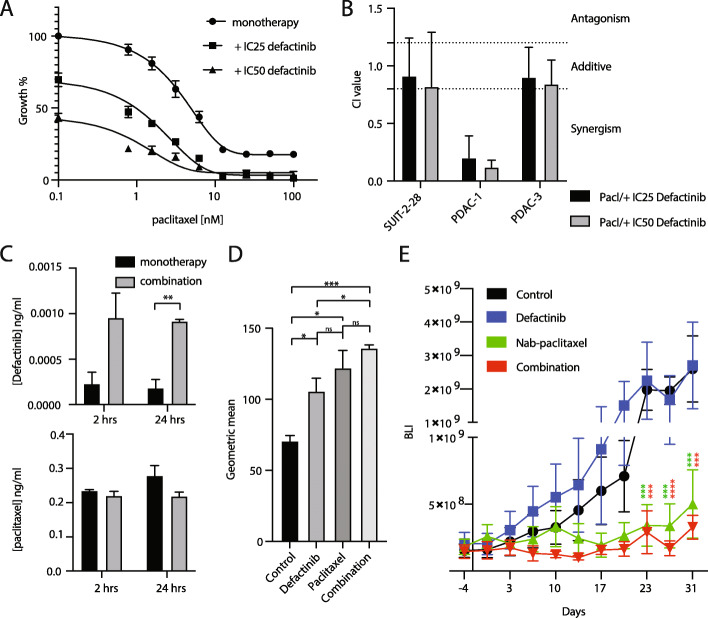
Combination treatment of defactinib with paclitaxel shows synergistic lethality in vitro and in vivo*.* **a.** Combination index was calculated after treatment of cells (Suit-2, PDAC-1, PDAC-3) with IC25 or IC50 of defactinib in combination with a range of paclitaxel dosing. Combination treatment showed strong synergistic effects in PDAC-1 and synergistic/additive effect in two other cell lines. **b.** Example of drug curves of primary cell line PDAC-1of defactinib in combination with paclitaxel. **c.** LC-MS measurement (*n* = 2) of drug concentration of cells (PDAC-1) exposed to defactinib/paclitaxel/combination showed highest intracellular concentrations of defactinib with combination. **d.** Polymerized tubulins were significantly highest in PDAC-1 cells treated with combination exposure to monotreatment. **e.** Growth of tumor size (defined as BLI in pancreas region) was significantly inhibited from day 23 to 31 in the nab-paclitaxel and combination group (multiple t-test, corrected for multiple testing by Holm-Sidak test)

Optimal treatment of PDAC currently exist of combinations of classical chemotherapeutics, such as gemcitabine in combination with nab-paclitaxel [2, 36, 37]. Combination treatment was evaluated in vitro for defactinib with either gemcitabine or paclitaxel (the unconjugated equivalent of nab-paclitaxel), drugs commonly used in PDAC therapy. Notably, combination therapy demonstrated strong synergy for the defactinib/paclitaxel combination in one cell line and synergistic/additive effect in the other two cell lines, as evident from a combination index score below 0.8 (Fig. 4a & b). However, combination of defactinib with gemcitabine did not enhance efficacy significantly (Supplemental Fig. 3E).

Intracellular drug concentrations were measured by LC-MS after 24 h of (co-)exposure with paclitaxel, which revealed an increase in defactinib concentrations intracellularly whilst paclitaxel concentration remained stable (Fig. 4c). Taxanes such as paclitaxel exercise their cytotoxic effect via stabilization of microtubule polymerization, which in turn inhibits cytoskeleton organization required for normal cellular functioning [38]. We evaluated the effect of combination effect on microtubules. For this, we measured polymerized microtubules by FACS analysis after exposure of vehicle, paclitaxel, defactinib or both drugs. Interestingly, PDAC cells showed induced polymerized microtubules upon treatment with either monotherapy. Additionally, combination treatment induced the highest polymerized microtubules (Fig. 4d), effectively increasing cellular toxicity. These results indicate that the increased treatment efficacy is most likely due to the higher defactinib drug availability intracellularly, possibly increasing synergistically targeting cytoskeleton organization and inhibiting cell division by hampering microtubule dynamics. These results highlight the synergistic potential of defactinib combination with paclitaxel in vitro.

### Combination of FAK inhibition with paclitaxel treatment results in reduced tumor growth in vivo

One primary cell line (PDAC-1) showed high sensitivity to FAK inhibition and the strongest synergy in vitro to the combination therapy, hence this cell line was used in an orthotopic grafting model. Early passages were used to retain original tumor features. After stratification to create groups of mice with comparable tumor inoculation, bioluminescence was observed in each group (*n* = 5 or 6 mice per arm, *p*-value non-significant, Supplemental Fig. 4A). Tumor growth was monitored during treatment. Defactinib monotreatment had modest efficacy in some mice, but overall had no effect on tumor size or growth. Nab-paclitaxel monotherapy was effective against tumor growth (Fig. 4e). The combination of nab-paclitaxel/ defactinib was well tolerated by the mice demonstrating efficacy and stable inhibition of tumor progression during treatment (Supplemental Fig. 4B). The study showed improved overall survival of mice treated with nab-paclitaxel and combination therapy compared to vehicle and defactinib monotherapy (Supplemental Fig. 4C). However, there was no difference on overall survival between the combination therapy and monotherapy with nab-paclitaxel, most probably because nab-paclitaxel was extremely effective in the anti-growth response in this mouse model, precluding optimal synergistical testing.

## Discussion

In this study, we identified FAK as a central player in a network of multiple phosphorylated tyrosine kinases in a phosphoproteome analysis of PDAC. Targeting FAK in vitro and in vivo reduced tumor growth and exhibited anti-tumor effects in combination with (nab-)paclitaxel.

Variance of the top ranked phosphorylated kinases was identified in the phosphoproteome screen; however, several kinases were identified in all cell lines, regardless of subtype of PDAC cell line. These results might be the result of the high penetrance of the driving *KRAS* mutation in these tumors [39] and lack of other activating mutations of oncogenes. Previous studies of our lab in different cancer types showed more heterogeneity in kinase ranking, affiliated by driver mutations [14, 40, 41]. Previously, Kim et al. [42] evaluated three primary cell lines from different metastatic origins of one patient and found clonal heterogeneity in PDAC, with a distinct (phospho)proteomic profiles of each metastatic site. However, their study did not reach the depth of the current phoshoproteome analysis. Interestingly, the focal adhesion pathway was likewise enriched in their data. Another study evaluated a panel of cell lines and primary cultures to distinguish phosphoproteomic subtypes. Upon multiple clustering steps, they highlighted a highly hyperphosphorylated subtype which was sensitive to erlotinib [43]. EGFR phosphorylation was indeed also identified in our cell line panel, however, targeting EGFR is currently not a regimen of preference for treatment of PDAC due to minimal clinical efficacy [44–46]. Other highly phosphorylated RTKs which were identified in our phosphoproteomic screen, including their down-stream regulatory proteins, were MET and EPHA2, two known modulators of oncogenic pathways and EGFR-resistant markers [47]. Additionally, these kinases have previously been established as possible targets by our group, validating the phosphoproteome approach [16, 48]. The enrichment of multiple targetable kinases opens the door for future combination studies of TKIs.

The target FAK identified here has recently gained interest as target in multiple solid cancers [28]. In PDAC, FAK inhibition showed an antiproliferative effect in vitro [49]. Importantly, FAK has been implicated as mediator in mechano-signaling in mice studies, exemplified by fibrotic density influencing pro-tumorigenic stimuli in PDAC through integrin B1 [50]. Additionally, FAK signaling was shown to mediate an immune-evasive tumor environment in genetic engineered mice models. Upon inhibition of FAK, these tumors were more sensitive to immune targeting therapy [51], prompting interest in FAK inhibition in combination with immunotherapy. Moreover, FAK inhibition affects cancer-associated fibroblast in the tumor microenvironment [52], further enhancing the functionality of targeting this kinase in PDAC patients.

These above-mentioned studies underline the potential of FAK inhibition on the tumor and microenvironment of PDAC, but synergistic combinations with established cytotoxic agents against PDAC have not been studied. Here, we show a synergistic effect of FAK inhibition by defactinib and (nab-)paclitaxel in vitro, but not with gemcitabine. Nab-paclitaxel is effective in penetrating the stroma of PDAC [53], and targets microtubules inducing polymerization. We have shown that the combination therapy induces an increase in the quantity of polymerized microtubules and thus enhances overall toxicity. Interestingly, in resistant ovarian cancer cells (another stroma inducing tumor), taxol resistance was overcome by FAK inhibition using PF-228 [54], hence supporting the logic of this combination. Moreover, an anti-migratory effect was observed in vitro, indicating an additional antitumor effect of defactinib treatment in PDAC.

Since PDAC is known for its heterocellular microenvironment, another study evaluated the influence of tumor-derived secreted proteins of PDAC cells and showed the complex bidirectional influence of the tumor cells on stromal signaling and vice versa in vitro [55]. One of the limitations of the current study is that the multicellular PDAC biology was not explored. Additionally, although synergistic effects were observed in vitro, smaller effects on growth inhibition were identified in vivo by combination therapy. Most likely, this is the result of the strong effect of monotherapy by nab-paclitaxel precluding synergistic studies. Also, mice did not receive a second round of treatment upon progression of disease, which hampers conclusions on efficacy on survival in this small cohort. These parameters will need to be further explored in future (pre)clinical studies. Moreover, we studied phosphotyrosine residues since their relevance for TKIs, but exploration of the full profile of cellular phosphorylation of tyrosine, theorine and serine residues could be informative for further elucidation of tumor biology. Novel studies performing integrated analysis of all levels of phosphorylation will deepen our knowledge of this disease. Moreover, future studies incorporating more complex tissue of patient-derived xenografts and patient samples will further enhance our knowledge of complex signaling wiring of PDAC.

## Conclusion

In conclusion, our study shows a high phosphorylation of several oncogenic receptor tyrosine kinases in PDAC cells and validated FAK inhibition as potential combination target with nab-paclitaxel in patient suffering from this devasting disease.

## Supplementary Information

**Additional file 1: Supplemental Fig. 1.** General description of phosphotyrosine-enriched analysis. **a**. Pearson correlation of technical replicates showed good correlation, replicate 1 represents a technical replicate in one dataset, while replicate 2 and 3 represent interexperiment replicates. **b**. Pie chart of all RTKs and non-RTKS identified in the discovery dataset show high coverage of the kinome. **c.** Unsupervised clustering of phosphorylated kinases identify an abundance of kinase activity. **Supplemental Fig. 2.** Evaluation of kinases and FAK in PDAC. **a.** Outlier analysis of kinases in the discovery dataset showed no outlier phosphorylation (Grubbs’ test, threshold 1%) in the top 10 phosphorylated kinases. Bar shows median with range. **b.** Phosphorylated FAK represents a significant quantity of total kinases identified. **c.** Sensitivity curves of VS-4718 and **d.** TAE226 confirms effectiveness of FAK inhibition in PDAC. (Error bars are SEM of biological replicates, *n* = 3). **Supplemental Fig. 3.** Defactinib is a potent FAK inhibitor. **a.** Unsupervised clustering of Suit-2 cells treated with vehicle or defactinib after two hours showed different clusters. **b.** Heatmap of INKA score upon treatment with defactinib. **c**. INKA score comparison of FAK activity **d.** Bar graph of significantly downregulated phosphopeptides (PP) of FAK substrates (Error bars are SD of biological replicates, *n* = 2, t-test * < 0.05, ** 0.01, *** < 0.001, **** < 0.001) **e.** Combination treatment of defactinib with gemcitabine did not induce synergy, but was additive or antagonistic. **Supplemental Fig. 4.** In vivo treatment of defactinib with nab-paclitaxel. **a.** Tumor induction expressed by BLI was equal in all groups after stratification at day 4 (bar depict averages with SEM, p = ns, Mann-Whitney test). **b**. Growth curves of tumor growth evaluated by BLI during first 14 days of treatment were evaluated by analysis of growth slope. Combination treatment slowed tumor growth the most (F-test for all slopes *p* < 0.0001)**. c.** Treatment with nab-paclitaxel or combination with defactinib improved overall survival (median survival: control 41.5 days, defactinib 56 days, nab-paclitaxel 77 days, combination 60 days).

## Data Availability

The mass spectrometry proteomics data will be deposited in the ProteomeXchange Consortium via the PRIDE [56] partner repository with the dataset identifier PXD024548 upon publication of the manuscript.

## Abbreviations

BLI: bioluminescence
CI: combination index
EGFR: epidermal growth factor receptor
EPHA2: eph-A2 receptor
ES: enrichment score
FAK: focal adhesion kinase
IC: inhibitory concentration
INKA: integrative inferred kinase activity
IV: intravenously
MET: hepatocyte growth factor receptor
PDAC: pancreatic ductal adenocarcinoma
Po: per os
PP: phosphopeptide
pY: phosphotyrosine
SRB: sulforhodamine B
ssGSEA: single sample gene set enrichment analysis
TKI: tyrosine kinase inhibitor
