# Supplementary material for: Focal adhesion kinase inhibition synergizes with nab-paclitaxel to target pancreatic ductal adenocarcinoma
Source: J Exp Clin Cancer Res. 2021 Mar 9;40:91. doi: 10.1186/s13046-021-01892-z (PMC7941981; doi:10.1186/s13046-021-01892-z)

Supplemental Figure 1

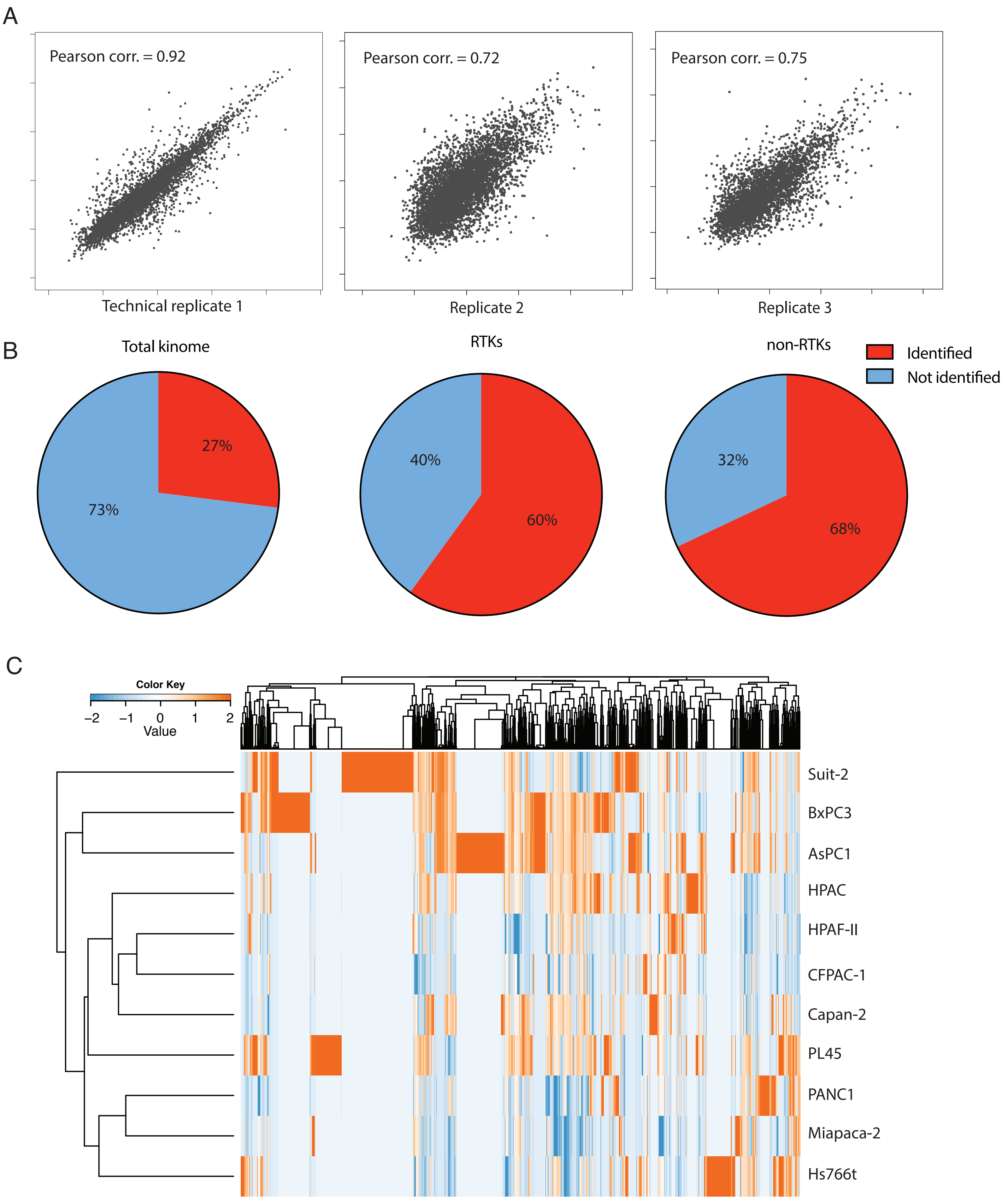

Supplemental Figure 2

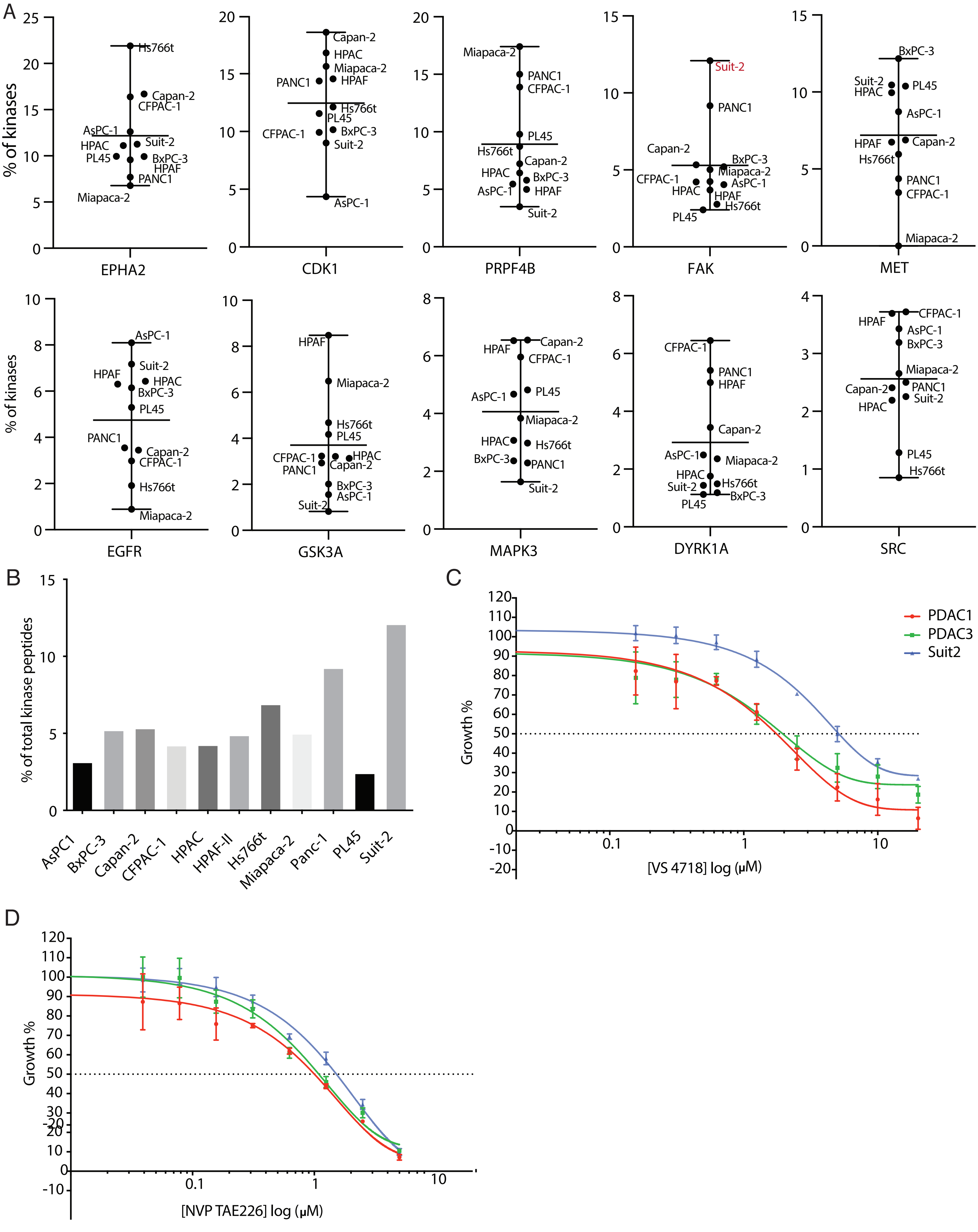

Supplemental Figure 3

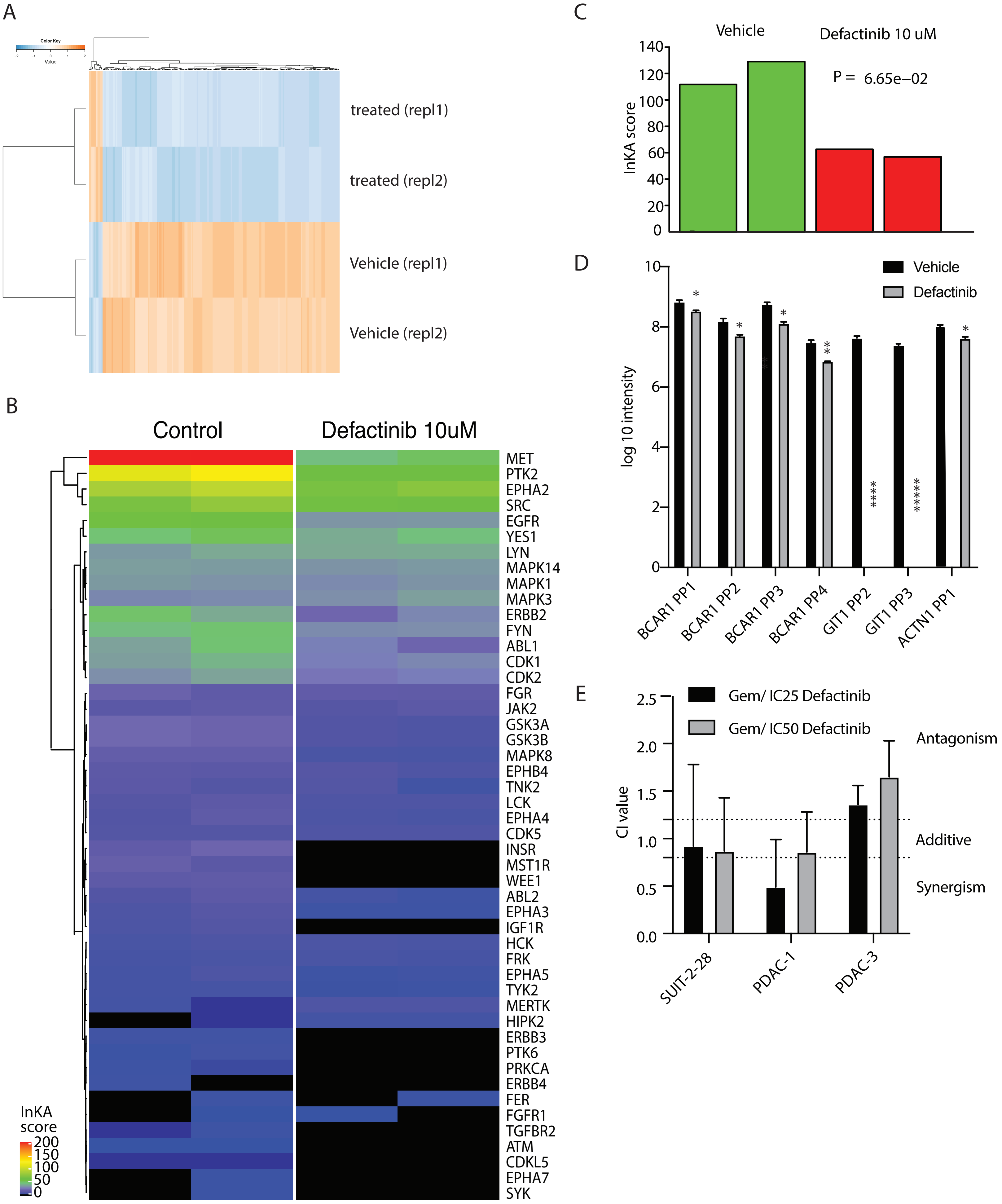

Supplemental Figure 4

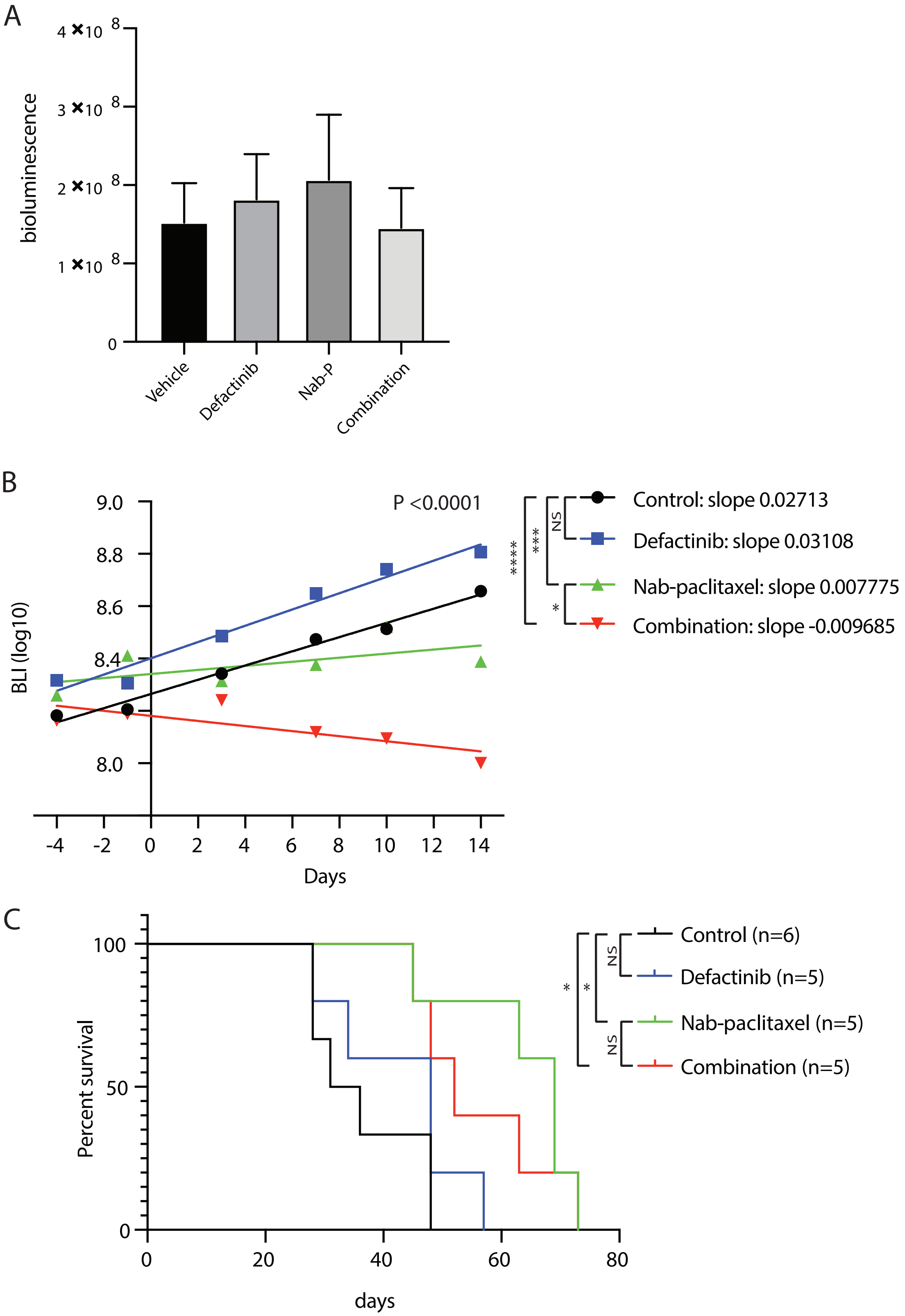

Supplement: Supplementary file 1 — Additional file 1: Supplemental Fig. 1. General description of phosphotyrosine-enriched analysis. a. Pearson correlation of technical replicates showed good correlation, replicate 1 represents a technical replicate in one dataset, while replicate 2 and 3 represent interexperiment replicates. b. Pie chart of all RTKs and non-RTKS identified in the discovery dataset show high coverage of the kinome. c. Unsupervised clustering of phosphorylated kinases identify an abundance of kinase activity. Supplemental Fig. 2. Evaluation of kinases and FAK in PDAC. a. Outlier analysis of kinases in the discovery dataset showed no outlier phosphorylation (Grubbs’ test, threshold 1%) in the top 10 phosphorylated kinases. Bar shows median with range. b. Phosphorylated FAK represents a significant quantity of total kinases identified. c. Sensitivity curves of VS-4718 and d. TAE226 confirms effectiveness of FAK inhibition in PDAC. (Error bars are SEM of biological replicates, n = 3). Supplemental Fig. 3. Defactinib is a potent FAK inhibitor. a. Unsupervised clustering of Suit-2 cells treated with vehicle or defactinib after two hours showed different clusters. b. Heatmap of INKA score upon treatment with defactinib. c. INKA score comparison of FAK activity d. Bar graph of significantly downregulated phosphopeptides (PP) of FAK substrates (Error bars are SD of biological replicates, n = 2, t-test * < 0.05, ** 0.01, *** < 0.001, **** < 0.001) e. Combination treatment of defactinib with gemcitabine did not induce synergy, but was additive or antagonistic. Supplemental Fig. 4. In vivo treatment of defactinib with nab-paclitaxel. a. Tumor induction expressed by BLI was equal in all groups after stratification at day 4 (bar depict averages with SEM, p = ns, Mann-Whitney test). b. Growth curves of tumor growth evaluated by BLI during first 14 days of treatment were evaluated by analysis of growth slope. Combination treatment slowed tumor growth the most (F-test for all slopes p < 0.0001). c. T [file 13046_2021_1892_MOESM1_ESM.pdf]
